# Supplementary material for: Observation of frustrated chiral dynamics in an interacting triangular flux ladder
Source: Nat Commun. 2023 Nov 20;14:7560. doi: 10.1038/s41467-023-43204-3 (PMC10662351; doi:10.1038/s41467-023-43204-3)
Supplement: Supplementary file 1 — Supplementary Information [file 41467_2023_43204_MOESM1_ESM.pdf]

Supplementary Information for "Observation of frustrated chiral dynamics in an interacting triangular flux ladder"

Yuqing Li[1,2], Huiying Du[1], Yunfei Wang[1], Junjun Liang[1],
Liantuan Xiao[1,2], Wei Yi[3,4,5], Jie Ma[1,2,5], Suotang Jia[1,2],
[1]State Key Laboratory of Quantum Optics and Quantum Optics Devices, Institute of Laser Spectroscopy, College of Physics and Electronics Engineering, Shanxi University, Taiyuan 030006, China
[2]Collaborative Innovation Center of Extreme Optics, Shanxi University, Taiyuan 030006, China
[3]CAS Key Laboratory of Quantum Information, University of Science and Technology of China, Hefei 230026, China
[4]CAS Center For Excellence in Quantum Information and Quantum Physics, Hefei 230026, China
[5]Hefei National Laboratory, Hefei 230088, China

In this Supplementary Information, we provide more details on the experimental realization of the triangular ladder, the mapping between the discrete momentum states and sites in the synthetic triangular ladder, the coupled Gross-Pitaevskii (GP) equations and the effective Hamiltonian, the chiral dynamics under different interactions, the ground-state phase diagram of a triangular ladder in the noninteracting limit, and the dynamic behaviors of a square flux ladder.

Supplementary Note 1
Experimental realization of the triangular flux ladder

Prepared by a hybrid evaporative cooling, the Bose-Einstein condensate (BEC) of ^{133}Cs atoms is confined to a cigar-shaped optical trap with the trap frequencies $(\omega_x, \omega_y, \omega_z) = 2\pi \times (125, 96, 13)$ Hz [1]. The trapping laser beam along the z-direction (with a wavelength of λ = 1064 nm) is retro-reflected to form a pair of counter-propagating lasers. In contrast to the incoming laser beam, whose frequency is fixed at ω, two acoustic optical modulators are used in the reflected laser beam to generate multi-frequency components $\omega_{m,m+1}$. These laser fields drive a series of two-photon Bragg transitions between the discrete atomic momentum states with an increment of $2\hbar k$ (with the reduced Planck's constant $\hbar$ and $k = 2\pi/\lambda$). The frequency difference coincides with the quadratic dispersion of free atoms, as ω - $\omega_{m,m+1}$ = $(2m+1)4E_R/\hbar$, and E_R is the atomic recoil energy. The two-photon Bragg transitions mimic, in the momentum space, the nearest-neighbor (NN) tunnelings in the synthetic one-dimensional (1D) lattice [2, 3].

We construct a triangular ladder by adding the next-nearest-neighbor (NNN) couplings in a 14-site 1D momentum lattice [4]. The NNN couplings are implemented through multiple four-photon Bragg transitions between the discrete momentum states with the increment of $4\hbar k$ (see Fig. 1b of the main text). We introduce additional frequency components $\omega_{m,m+2}$ in the reflected laser beam for these four-photon Bragg transitions. The frequency difference is fixed by ω - $\omega_{m,m+2} = \frac{1}{2}\left\{(2m+1) + \left[2(m+1)+1\right]\right\} 4E_R/\hbar$ [4, 5].

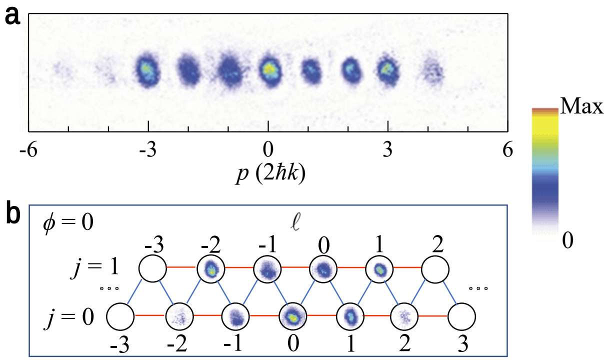

Supplementary Figure 1. Mapping between the experimentally measured one-dimensional momentum distribution and the density distribution along the synthetic ladder. **a**, Image of the atomic one-dimensional momentum distribution taken after the 600 μs evolution and 22-ms time-of-flight. **b**, The atomic density distribution in the ladder is constructed by rearranging the atomic population distribution in **a** according to the mapping relation $m = 2\ell + j$ between the momentum state $p = 2m\hbar k$ and the site (ℓ, j).

The synthetic sites based on the laser-coupled momentum states are then mapped to those along a triangular ladder with 2 × 7 sites, as illustrated in Fig. 1c of the main text, where the NN and NNN couplings correspond to the inter- and intra-leg couplings, respectively. By controlling the amplitude of each multi-frequency component, all NN and NNN couplings can be individually addressed. The actual tunneling energies are frequently calibrated by using the two-site Rabi oscillations to suppress the fluctuations below 5 %. To implement the phase ϕ in the NNN hoppings, we apply the controllable phase to the laser component with frequency $\omega_{m,m+2}$, with respect to the incoming single-frequency laser beam.

Supplementary Note 2
Atomic density distribution in the ladder

The dynamics of atoms in the synthetic ladder can be probed by measuring the evolution of density distri-

bution, where all atoms are initialized in the site (0, 0). In Supplementary Figure 1, we show an example on obtaining the density distribution of atoms in the synthetic ladder with zero flux in the noninteracting regime. After an evolution time of $t = 600$ μs, we switch off all laser fields, and detect the population distribution of atoms in different momentum states after a 22-ms time-of-flight via the standard absorption imaging technique as shown in Supplementary Figure 1a. The atomic density distribution in the ladder, as shown in Supplementary Figure 1b, is then constructed by mapping the momentum state $p = 2m\hbar k$ to the lattice site (ℓ, j) in the synthetic ladder, using the relation $m = 2\ell + j$.

Supplementary Note 3

GP equations and the effective Hamiltonian

In this section, we discuss in detail the coupled GP equations that we use for the numerical calculations, as well as the underlying second-quantized Hamiltonian.

The momentum lattice used to synthesize the triangular ladder is based on a series of Bragg transitions between the discrete momentum states of the optically trapped BEC. The field consisting of the Bragg laser pairs along the z-direction is described by

$$\varepsilon = \frac{1}{2}\hat{\varepsilon}_0 e^{i(k_0 z - \omega_0 t + \tilde{\phi}_0)} + \sum_l \frac{1}{2}\hat{\varepsilon}_1 e^{i(-k'_l z - \omega'_l t + \phi'_l)} + \sum_l \frac{1}{2}\hat{\varepsilon}_2 e^{i(-k_l z - \omega_l t + \phi_l)} + \text{c.c.}, \quad (1)$$

where $\hat{\varepsilon}_0$ is the electric field of the incident laser, $\hat{\varepsilon}_1$ ($\hat{\varepsilon}_2$) is the electric field of the retro-reflected laser used to realize the NN (NNN) couplings in the ladder, and the wave vectors are approximately the same with $k_0 \simeq k'_l \simeq k_l = k$.

The Bragg lasers are far-off-resonant, so that we adiabatically eliminate the excited states [6, 7], and the interactions of the Bragg laser beams with atoms lead to two kinds of time-dependent lattice potentials on the ground state

$$V_{\text{NN}}(z,t) = \sum_l 2J\cos(2kz - \delta_l t + \phi'_l - \tilde{\phi}_0), \quad (2)$$

$$V_{\text{NNN}}(z,t) = \sum_l 2K\cos(4kz - \xi_l t + \phi_l - \tilde{\phi}_0), \quad (3)$$

where the $J = \hbar\Omega_0\Omega_1/4|\Delta|$ and $\delta_l = \omega_0 - \omega'_l$ are the strength and detuning of the NN couplings, and $K = \hbar\Omega_0\Omega_2/4|\Delta|$ and $\xi_l = \omega_0 - \omega_l$ are the strength and detuning of the NNN couplings. Here $\Omega_{0,1,2}$ are respectively the single-photon Rabi-frequencies under the electric fields $\hat{\varepsilon}_{0,1,2}$.

For a BEC in the combined presence of a harmonic trapping potential V_{trap} and the optical potentials of the Bragg lasers V_{NN} and V_{NNN}, its wave function $\Psi(\mathbf{r},t)$ obeys the GP equation

$$i\hbar\frac{\partial\Psi}{\partial t} = \Big(-\frac{\hbar^2\nabla^2}{2M} + V_{\text{trap}} + V_{\text{NN}} + V_{\text{NNN}} + \frac{4\pi\hbar^2 a}{M}N|\Psi|^2\Big)\Psi \quad (4)$$

with the normalization condition $\int d\mathbf{r}|\Psi(\mathbf{r},t)|^2 = 1$. Here, M is the mass of ^{133}Cs atom, a is the atomic s-wave scattering length and N is the total atom number. By writing down the GP equation, we have already taken the mean-field approximation.

Focusing on the discrete set of momentum modes coupled by the Bragg transitions, we use the ansatz $\Psi(\mathbf{r},t) = \sum_m \psi_m(\mathbf{r},t)e^{2imkz}$ to decompose the wavefunction into discrete momentum components [8, 9], where ψ_m is the wave function of the mth momentum mode with a momentum $p = 2m\hbar k$ and $m \in \mathbb{Z}$. It follows that the coupled GP equations for ψ_m are

$$\begin{aligned} i\hbar\frac{\partial\psi_m}{\partial t} = &\Big(-\frac{2\hbar mk}{M}i\hbar\partial_z + \frac{4m^2k^2\hbar^2}{2M} - \frac{\hbar^2}{2M}\nabla^2 + V_{\text{trap}}\Big)\psi_m \\ &+ \sum_l J(e^{i(-\delta_l t + \phi'_l - \tilde{\phi}_0)}\psi_{m-1} + e^{-i(-\delta_l t + \phi'_l - \tilde{\phi}_0)}\psi_{m+1}) \\ &+ \sum_l K(e^{i(-\xi_l t + \phi_l - \tilde{\phi}_0)}\psi_{m-2} + e^{-i(-\xi_l t + \phi_l - \tilde{\phi}_0)}\psi_{m+2}) \\ &+ \frac{4\pi\hbar^2 a}{M}N\Big(|\psi_m|^2 + \sum_{\kappa\neq m} 2|\psi_\kappa|^2\Big)\psi_m. \end{aligned} \quad (5)$$

Here the inter-atomic interactions give rise to density-dependent potentials in the last line. Note that we have neglected scattering processes involving more than two momentum modes, since they are energetically suppressed compared to the density-dependent terms that we retain. Alternatively, this can be understood as a Hartree-Fock-like approximation in momentum lattices.

In our experiment, the synthetic flux is produced by engineering the tunneling phase ϕ_l in the NNN couplings as shown in Fig. 1c of the main text, while $\tilde{\phi}_0 = \pi$ and $\phi'_l = 0$. Under the transformation (that is, choosing a proper rotating frame) $\varphi_m(\mathbf{r},t) = e^{i4m^2E_r t/\hbar}\psi_m(\mathbf{r},t)$, with $E_r = \hbar^2k^2/(2M)$, Eq. (5) becomes

$$\begin{aligned} i\hbar\frac{\partial\varphi_m}{\partial t} = &\Big(-\frac{2\hbar mk}{M}i\hbar\partial_z - \frac{\hbar^2}{2M}\nabla^2 + V_{\text{trap}}\Big)\varphi_m \\ &- \sum_l Je^{i(-\delta_l t + 4(2m-1)\frac{E_r}{\hbar}t)}\varphi_{m-1} \\ &- \sum_l Je^{-i(-\delta_l t + 4(2m+1)\frac{E_r}{\hbar}t)}\varphi_{m+1} \\ &- \sum_l Ke^{i(-\xi_l t + \phi_l + 8(2m-2)\frac{E_r}{\hbar}t)}\varphi_{m-2} \\ &- \sum_l Ke^{-i(-\xi_l t + \phi_l + 8(2m+2)\frac{E_r}{\hbar}t)}\varphi_{m+2} \\ &+ \frac{4\pi\hbar^2 a}{M}N\Big(|\varphi_m|^2 + \sum_{\kappa\neq m} 2|\varphi_\kappa|^2\Big)\varphi_m, \end{aligned} \quad (6)$$

where $\delta_l = 4(2l+1)E_r/\hbar$ and $\xi_l = 8(2l+2)E_r/\hbar$. Neglecting the off-resonant Bragg processes, we reduce Eq. (6) to

$$\begin{aligned} i\hbar\frac{\partial\varphi_m}{\partial t} =& \Big(-\frac{2\hbar mk}{M}i\hbar\partial_z - \frac{\hbar^2}{2M}\nabla^2 + V_{\text{trap}}\Big)\varphi_m \\ &- J\varphi_{m+1} - J\varphi_{m-1} \\ &- Ke^{-i\phi_m}\varphi_{m+2} - Ke^{i\phi_{m-2}}\varphi_{m-2} \\ &+ \frac{4\pi\hbar^2 a}{M}N\Big(|\varphi_m|^2 + \sum_{\kappa\neq m}2|\varphi_\kappa|^2\Big)\varphi_m. \end{aligned} \quad (7)$$

Under a weak harmonic trapping potential, we assume that each momentum mode features a homogeneous density distribution, with $\varphi_m = \tilde{\varphi}_m/\sqrt{V_0}$ (V_0 being the quantization volume). The above equation can then be written as

$$\begin{aligned} i\hbar\frac{\partial\tilde{\varphi}_m}{\partial t} =& -J\tilde{\varphi}_{m+1} - J\tilde{\varphi}_{m-1} \\ &- Ke^{-i\phi_m}\tilde{\varphi}_{m+2} - Ke^{i\phi_{m-2}}\tilde{\varphi}_{m-2} \\ &+ U\Big(|\tilde{\varphi}_m|^2 + \sum_{\kappa\neq m}2|\tilde{\varphi}_\kappa|^2\Big)\tilde{\varphi}_m, \end{aligned} \quad (8)$$

where the $\tilde{\varphi}_m$ fulfils the normalization condition $\sum_m|\tilde{\varphi}_m|^2 = 1$, and the mean-field interaction energy is $U = (4\pi\hbar^2 a/M)\rho$ with the average density $\rho = N/V_0$.

Mapping the coupled GP equations from the discrete momentum states to sites in the synthetic ladder, we have

$$\begin{aligned} i\hbar\frac{\partial\tilde{\varphi}_{\ell,j}}{\partial t} =& -J\tilde{\varphi}_{\ell,j+(-1)^j} - J\tilde{\varphi}_{\ell-(-1)^j,j+(-1)^j} \\ &- Ke^{-i\phi_{\ell,j}}\tilde{\varphi}_{\ell+1,j} - Ke^{i\phi_{\ell-1,j}}\tilde{\varphi}_{\ell-1,j} \\ &+ U\Big(|\tilde{\varphi}_{\ell,j}|^2 + \sum_{j'\neq j}2|\tilde{\varphi}_{\ell,j'}|^2 + \sum_{\ell'\neq\ell}2|\tilde{\varphi}_{\ell',j}|^2 \\ &+ \sum_{\ell'\neq\ell,j'\neq j}2|\tilde{\varphi}_{\ell',j'}|^2\Big)\tilde{\varphi}_{\ell,j}. \end{aligned} \quad (9)$$

This is the coupled GP equation that we used for all numerical calculations. Based on Eq. (9), we can read off the second-quantized Hamiltonian

$$\begin{aligned} H =& -\Big[J\sum_\ell(\hat{c}^\dagger_{\ell,1}\hat{c}_{\ell,0} + \hat{c}^\dagger_{\ell,1}\hat{c}_{\ell+1,0}) + K\sum_{\ell,j}e^{i(-1)^j\phi}\hat{c}^\dagger_{\ell+1,j}\hat{c}_{\ell,j} \\ &- \text{h.c.}\Big] + \frac{U}{2N}\sum_{\ell,j}n_{\ell,j}(n_{\ell,j}-1) \\ &+ \frac{2U}{N}\left(\sum_{j'<j}\sum_{\ell,\ell',j}n_{\ell',j'}n_{\ell,j} + \sum_{\ell'<\ell}\sum_{\ell,j}n_{\ell',j}n_{\ell,j}\right), \end{aligned} \quad (10)$$

where $\hat{c}^\dagger_{\ell,j}$ and $\hat{c}_{\ell,j}$ are the creation and annihilation operators for atoms with site label (ℓ,j). The number operator is $n_{\ell,j} = \hat{c}^\dagger_{\ell,j}\hat{c}_{\ell,j}$. Considering the conservation of total atom number $\sum_{\ell,j}n_{\ell,j} = N$, the interaction terms in Hamiltonian (10) can be simplified as

$$H_{\text{int}} = U(N-\frac{1}{2}) - \frac{U}{2N}\sum_{\ell,j}n^2_{\ell,j}. \quad (11)$$

Alternatively, one may start from a second-quantized Hamiltonian in the real space. Assuming that only the discrete momentum modes coupled by the Bragg transitions are relevant (neglecting the weak harmonic trap), and taking the Hartree-Fock-like approximation in the resulting momentum lattice, one would arrive at the second-quantized Hamiltonian (10). Then, taking the mean-field approximation $\langle c_{\ell,j}\rangle = \tilde{\varphi}_{\ell,j}$ in the Heisenberg equations of motion of the field operators $c_{\ell,j}$, one gets the coupled GP equations in Eq. (9).

Supplementary Note 4

Interaction effects on the chiral dynamics along the triangular flux ladder

In Supplementary Figure 2, we show the experimentally measured time evolution of the average site index and inter-leg polarization under different fluxes and interactions. In Supplementary Figures 2a-c, the dynamical evolution of average site index $\langle\ell\rangle_0$ is measured in units of the tunneling time $\hbar/J \approx 400$ µs for three different interaction strengths. In all cases, a change in sign of the flux reverses the atomic motion, a central feature of the chiral dynamics. With increasing interaction strength U/J, however, the dynamics becomes more and more localized to the initial site. This is a direction manifestation of the interaction-induced self-trapping. The experimental data are in good agreement with the numerical simulations (solid lines).

In Supplementary Figures 2d-f, we show the impact of interactions on the time evolution of the inter-leg polarization $\Delta P = P_0(t)$ - $P_1(t)$. In the noninteracting regime, the polarization exhibits a damped oscillation centered near a small polarization. Here the decoherence between atoms in different momentum states limits the time of observation. With increasing interaction strength U/J, the oscillations become centered toward larger polarizations, indicating the emergence of a biased dynamics in between the two legs.

Supplementary Note 5

Ground-state phase diagram of a noninteracting triangular flux ladder

In this section, we present the ground-state phase diagram of a noninteracting triangular flux ladder, and discuss its relation with the system dynamics.

We start from the single-particle Hamiltonian

$$\begin{aligned} H =& -J\sum_\ell(\hat{a}^\dagger_\ell\hat{b}_\ell + \hat{b}^\dagger_\ell\hat{a}_{\ell+1} + \text{h.c.}) \\ &- K\sum_\ell(e^{i\phi}\hat{a}^\dagger_\ell\hat{a}_{\ell+1} + e^{-i\phi}\hat{b}^\dagger_\ell\hat{b}_{\ell+1} + \text{h.c.}), \end{aligned} \quad (12)$$

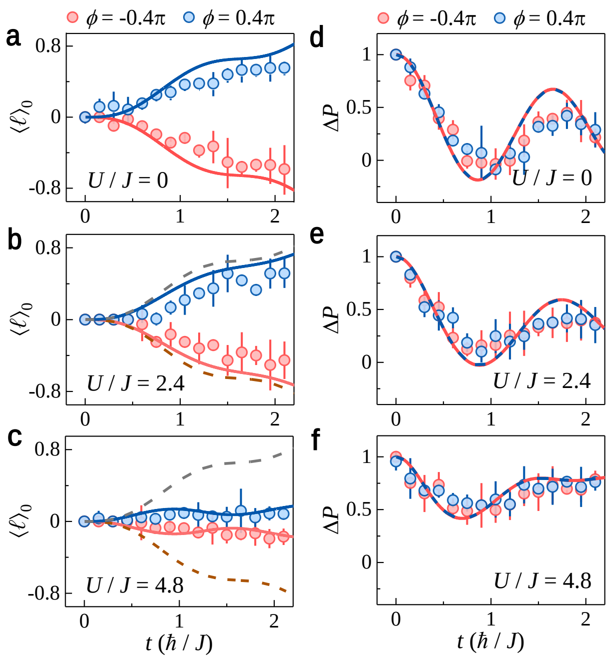

Supplementary Figure 2. Interaction effects on the chiral dynamics. Dynamical evolutions of average site index $\langle \ell \rangle_0$ in the $j = 0$ leg in **a-c** and inter-leg polarization ΔP in **d-f** with the fluxes $\phi = \pm 0.4\ \pi$ for three different interactions $U/J = 0$, 2.4 and 4.8. The solid and dashed lines are the results from numerical simulations in **a-c** and **d-f**, respectively. The dashed lines in **a-c** show the single-particle predictions. All error bars denote standard errors. In all panels, the inter- and intra-leg hopping rates are $J/h = 0.4$ kHz and $K/h = 0.2$ kHz, respectively.

where $\hat{a}_\ell^\dagger$ ($\hat{b}_\ell^\dagger$) and $\hat{a}_\ell$ ($\hat{b}_\ell$) are the creation and annihilation operators for atoms in the $j = 0$ ($j = 1$) leg, respectively. To diagonalize the Hamiltonian (12), we perform Fourier transformations $\hat{a}_\ell = \frac{1}{\sqrt{L}} \sum_k e^{ik\ell} \hat{a}_k$ and $\hat{b}_\ell = \frac{1}{\sqrt{L}} \sum_k e^{ik\ell} \hat{b}_k$ [10, 11], where L represents the number of synthetic sites in each leg, and the quasimomentum k lies within the first Brillouin zone $-\pi < ka \leqslant \pi$. In the following, we set the lattice constant to $a = 1$. Defining the spinor operator $\hat{\Psi}_k \equiv \begin{bmatrix} \hat{a}_k \\ \hat{b}_k \end{bmatrix}$, the noninteracting Hamiltonian in the reciprocal space takes the form $H = \sum_k \hat{\Psi}_k^\dagger H(k) \hat{\Psi}_k$, where

$$H(k) = -2K \begin{bmatrix} \cos(k+\phi) & \xi\left(1+e^{-ik}\right) \\ \xi\left(1+e^{ik}\right) & \cos(k-\phi) \end{bmatrix}, \quad (13)$$

and $\xi = J/(2K)$. The eigenspectrum is given by

$$E_\pm(k) = -2K\cos(\phi)\cos(k) \pm \sqrt{4K^2\sin^2(\phi)\sin^2(k) + 2J^2(1+\cos(k))}, \quad (14)$$

which describes the two-band structure.

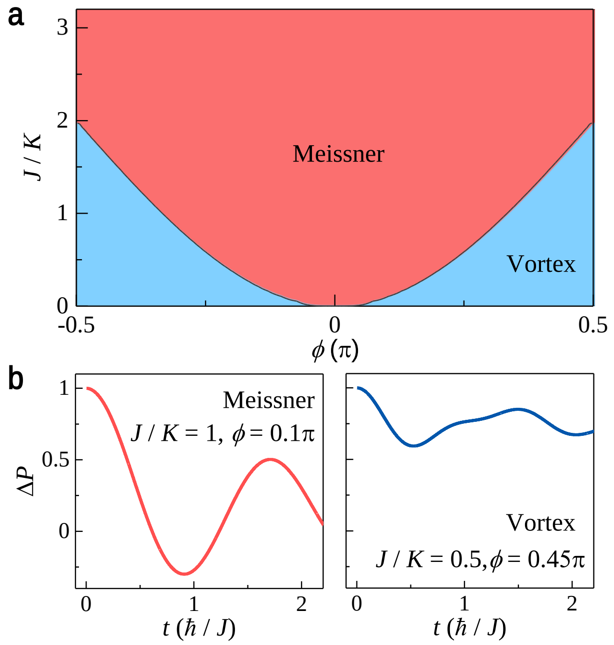

Supplementary Figure 3. The triangular flux ladder in the noninteracting limit. **a**, The ground-state phase diagram. The phase boundary is calculated according to Eq. (15). **b**, The numerically simulated time evolution of the inter-leg polarization $\Delta P = P_0(t)$ - $P_1(t)$ for different J/K and ϕ.

To understand the ground state, we focus on the lower band with E_-. When $J/K > (J/K)_c$, the lower band has a single minimum at $k = 0$. This is similar to the Meissner phase in a square flux ladder [12–15]. When $J/K < (J/K)_c$, the lower band has two degenerate minima located at $k = \pm k_0$ with $k_0 > 0$, similar to the vortex phase in a square flux ladder. The two phases are separated by the flux-dependent boundary

$$\sin^4(\phi) = \frac{1}{2}(J/K)_c^2\left[1+\cos^2(\phi)\right] - \frac{1}{16}(J/K)_c^4. \quad (15)$$

In Supplementary Figure 3a, we show the ground-state phase diagram of the triangular flux ladder in the noninteracting limit according to Eq. (15). In Supplementary Figure 3b, we also show the numerical time-evolved inter-leg polarizations under the parameters of the two phases, where all atoms are initialized in the site (0, 0). Apparently, the time-averaged polarization is much closer to zero when the ground state is in the Meissner phase, whereas the polarization is closer to 1 in the vortex phase. This suggests that, while dynamics in our experiment typically involves multiple eigenstates, they can also reflect ground-state properties. In Fig. 1g of the main text, the visible increase in the polarization near $\phi \sim \pm\pi/4$ and $U/J \sim 0$ is therefore related to the ground-state phase transition.

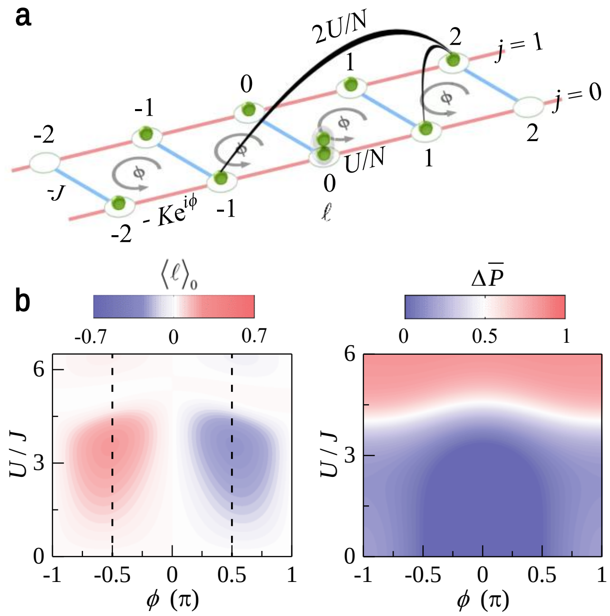

Supplementary Figure 4. Momentum-space square two-leg ladder and regimes of distinct dynamic behaviors. **a**, Schematic of a square two-leg ladder with the inter- and intra-leg hopping rates J and K, respectively. The applied tunneling phases in the $j = 0$ leg generate the flux ϕ in each four-site plaquette. As similar to the interaction of atoms in the triangular ladder, atoms in the same site experience an interaction strength U/N (grey shadow), and atoms in different sites have an interaction strength $2U/N$ (black lines). **b**, Left: the variation of average site index $\langle \ell \rangle_0$ in the $j = 0$ leg at the evolution time $t = 1.5$ $\hbar/J$ with the flux ϕ and interaction U/J. The red and blue regions represent the propagation of atoms along the $j = 0$ leg with the opposite directions, while the localized phase in the white region. Right: the average inter-leg polarization $\Delta\overline{P}$ within the time $t = 2$ $\hbar/J$ as a function ϕ and U/J. The blue and red regions represent the equal population of atoms between two legs and the localization of atoms in the $j = 0$ leg, respectively.

Supplementary Note 6

Dynamics in a square flux ladder

In this section, to reveal the impact of the frustrated geometry of a triangular ladder, we numerically study the dynamic behaviors of a square flux ladder under experimentally relevant parameters. In Supplementary Figure 4a, we show the configuration of the square ladder consisting of 2×7 sites with the homogeneous flux and controllable atomic interactions. For numerical simulations, we initialize the condensate in the site (0, 0), and numerically evolve the wave function to calculate the average site index and time-averaged inter-leg polarizations, which are shown in Supplementary Figure 4b.

Compared with Fig. 1g of the main text, the dynamics in the square ladder are different in two essential ways. First, the biased chiral regions at intermediate flux are absent in the square ladder. Instead, the average inter-leg polarizations are either close to zero or unity. Second, the persistent balanced chiral regions near $\phi = \pm\pi$ (and particularly at large interactions) are absent in the square ladder. The dynamics always becomes localized (in both directions) under a strong enough interaction. We attribute these behaviors in the synthetic triangular ladder to the interplay of interaction, flux and the frustrated geometry.

Supplementary References

[1] Wang, Y. F. et al. Hybrid evaporative cooling of ^{133}Cs atoms to Bose-Einstein condensation. Opt. Express 29, 13960 (2021).

[2] Meier, E. J., An, F. A. & Gadway, B. Atom-optics simulator of lattice transport phenomena. Phys. Rev. A 93, 051602(R) (2016).

[3] Wang, Y. F. et al. Observation of interaction-induced mobility edge in an atomic Aubry-André wire. Phys. Rev. Lett. 129, 103401 (2022).

[4] An, F. A., Meier, E. J. & Gadway, B. Engineering a flux-dependent mobility edge in disordered zigzag chains. Phys. Rev. X 8, 031045 (2018).

[5] Li, Y. Q. et al. Atom-optically synthetic gauge fields for a noninteracting Bose gas. Light Sci. Appl. 11, 13 (2022).

[6] Blakie, P. B. & Ballagh, R. J. Mean-field treatment of Bragg scattering from a Bose-Einstein condensate. J. Phys. B: At. Mol. Opt. Phys. 33, 3961 (2000).

[7] Pötting, S., Cramer, M. & Meystre, P. Momentum-state engineering and control in Bose-Einstein condensates. Phys. Rev. A 64, 063613 (2001).

[8] Dalfovo, F., Giorgini, S., Pitaevskii, L. P. & Stringari, S. Theory of Bose-Einstein condensation in trapped gases. Rev. Mod. Phys. 71, 463 (1999).

[9] Chen, T., Xie, D. Z., Gadway, B. & Yan, B. A Gross-Pitaevskii-equation description of the momentum-state lattice: roles of the trap and many-body interactions. arXiv:2103.14205v2 (2021).

[10] Uchino, S. & Tokuno, A. Population-imbalance instability in a Bose-Hubbard ladder in the presence of a magnetic flux. Phys. Rev. A 92, 013625 (2015).

[11] Halati, C. -M. & Giamarchi, T. Bose-Hubbard triangular ladder in an artificial gauge field. Phys. Rev. Research 5, 013126 (2023).

[12] Orignac, E. & Giamarchi, T. Meissner effect in a bosonic ladder. Phys. Rev. B 64, 144515 (2001).

[13] Piraud, M. et al. Vortex and Meissner phases of strongly interacting bosons on a two-leg ladder. Phys. Rev. B 91, 140406(R) (2015).

[14] Petrescu, A. & Hur, K. L. Chiral Mott insulators, Meissner effect, and Laughlin states in quantum ladders. Phys. Rev. B 91, 054520 (2015).

[15] Qiao, X. et al. Quantum phases of interacting bosons on biased two-leg ladders with magnetic flux. Phys. Rev. A 104, 053323 (2021).
